# Prevalence, pain intensity and symptoms associated with primary dysmenorrhea: a cross-sectional study

**DOI:** 10.1186/s12905-023-02878-z

**Published:** 2024-02-04

**Authors:** Jordana Barbosa-Silva, Mariana Arias Avila, Raissa Fernanda de Oliveira, Anny Caroline Dedicação, Amanda Garcia Godoy, Jessica Cordeiro Rodrigues, Patricia Driusso

**Affiliations:** 1 Women’s Health Research Laboratory (LAMU), Department of Physical Therapy, Federal University of São Carlos, São Carlos, Brazil; 2 Laboratory of Research on Electrophysical Agents (LAREF), Physical Therapy Department, Federal University of São Carlos, São Carlos, Brazil; 3 Multiprofessional Residency Program, Hospital Israelita Albert Einstein (HIAE), São Paulo, Brazil

**Keywords:** Pelvic pain, Menstruation disturbances, Menstrual symptoms, Menstrual pain

## Abstract

**Background:**

Primary dysmenorrhea (PD) is an etiological cyclic pelvic pain related to the menstrual period; it can negatively impact women’s quality of life and productivity. The aim of the present study was to estimate the prevalence of PD and analyze associated symptoms in Brazilian women.

**Methods:**

An online cross-sectional study was carried out in Brazil, with a structured questionnaire regarding dysmenorrhea and associated symptoms. PD intensity was measured with the Numerical Rating Scale for Pain and classified as mild (1–3), moderate (4–7) and severe (> 8). The association between qualitative variables was performed using Pearson’s Chi-Square Test. The quantification of this association was measured using multinomial logistic regression models, with calculation of Odds Ratio and confidence interval. A significance level of 5% was considered.

**Results:**

A total of 10,070 women were included. Most participants classified PD intensity as moderate (40.4%, 41.9% and 49.7%) and severe (21.2%, 24.8% and 28.4%) in the previous month, 3 months and 5 years, respectively. The most common symptoms associated with PD were irritability, abdominal distension sensation, anxiety and feeling more emotional. The increased of the risk (OR > 1.0) for moderate and severe PD-related pain intensity is related to age, nulliparity and presence PD since adolescence.

**Conclusion:**

There is a high prevalence of PD among Brazilian women, and the most common symptoms reported were irritability, abdominal distension sensation, anxiety and feeling more emotional.

**Supplementary Information:**

The online version contains supplementary material available at 10.1186/s12905-023-02878-z.

## Background

Dysmenorrhea is defined as cyclic pelvic pain related to the menstrual period. It can be classified as primary dysmenorrhea (PD) (without associated pelvic or gynecological disease) [1] or secondary (due to associated pelvic conditions such as endometriosis, adenomyosis, diseases pelvic inflammatory disorders) [2]. The World Health Organization considers it the most important factor related to chronic pelvic pain [3]. The pathophysiology of PD is still unknown; however, it may be explained by the increase in the synthesis and release of prostaglandins during menstruation, which leads to hypercontractility of the myometrium, uterine muscle ischemia [4], hypoxia, pain [4, 5], and decreased pain threshold [5].

It is known that PD affects adolescent girls and women, with prevalence that ranges from 70 to 90% [6, 7], with 2–29% of women reporting severe PD-related pain [8, 9]. Previous studies have reported some associated factors, including heavy and more prolonged menstrual bleeding, younger age, nulliparity, irregular menstrual cycle, and a family history of dysmenorrhea [1, 9].

Although most studies found that PD-related pain intensity varies from moderate to severe [10], women often consider the pain a common characteristic of the menstrual cycle and do not report it [11] and/or do not seek medical care [7, 12]. However, pain intensity may directly impact the women’s daily activities, as it could be considered debilitating and result in absence from school or work [13, 14]. PD could lead to lower academic performance in adolescents and poor sleep quality, adversely affecting mood, causing anxiety, depression, and stress [1, 15], and an increased chance of having central sensitivity symptoms [16]. Therefore, PD has always been related to socioeconomic and social factors increasing healthcare costs and reducing individuals’ productivity [9].

Previous studies already reported the prevalence and symptoms associated with PD in Europe [17–19], Middle East [20–23], Africa [24, 25], North America [26, 27], and Asia [28–30]. Considering South America, the prevalence of PD in Brazilian women has been reported by two different studies. However, only women from Brazil’s Northeast region participated in the studies [31, 32]. It is known that Brazil has continental dimensions and a culturally heterogeneous territory, a fact that may contribute to differences in studies conducted in different parts of the country and worldwide. In addition, conditions such as healthcare access and public policies on healthcare issues may differ in Brazil compared to other sub-developed or developed countries, potentially impacting the perception of PD. Therefore, this study aimed to investigate the prevalence, pain intensity, and symptoms associated with PD among Brazilian women nationwide.

## Methods

### Study design and setting of the study

This cross-sectional study was developed by the Women’s Health Research Laboratory (LAMU) in the Physical Therapy Department at the Federal University of São Carlos and approved by the Ethics and Research Committee on Human Beings (CAAE: 29747120.0.000.5504). The present study was conducted following the Declaration of Helsinki.

The study’s disclosure and data collection occurred between July and September 2020 on social networks and online interaction platforms (Instagram, Facebook, and WhatsApp), aiming to reach women from all Brazilian states. The study was conducted remotely through Google’s online forms platform (Google Forms). Women had to accept the Informed Consent Form to participate in the research.

Women aged ≥ 18 years old and who had menses during the previous three months were included in the study. Pregnant women and participants who did not menstruate the previous year or reported health conditions (such as endometriosis and myoma) related to secondary dysmenorrhea were excluded.

### Sample size calculation

The sample size was determined using the G*Power software. Considering the data available from the Brazilian Institute of Geography and Statistics, in 2010, Brazil had about 51 million women of reproductive age (from 15 to 54 years old) living in the country. In order to obtain a representative sample, considering a sampling error of 1%, a confidence interval of 95%, and the prevalence of dysmenorrhea of 91% [9], the estimated sample was 9603 valid responses.

### Data collection and study instruments

Data collection was conducted by filling a self-administered semi-structured online instrument developed with the modified Delphi method to formulate the tool with questions based on previous knowledge described in the literature about PD. Subsequently, the questionnaire was sent to 15 Women’s Health specialist professionals for review. The questionnaire was adapted according to the professionals’ suggestions. After this, the questionnaire was returned to those professionals for further correction. This process was repeated until a consensus was reached [33]. The target population reviewed the questionnaire four times (35 menstruating women per round) to verify the adequacy of the language, the online format and the amount of time spent completing the questionnaire. In each step, the research group discussed the women’s suggestions, and the questionnaire was changed when relevant. The questionnaire included questions related to sociodemographic characteristics (e.g., region in which the participants lived, educational level, age and marital status), gynecological and obstetric data (e.g., number of pregnancies and use of hormonal contraceptives), and questions related to the menstrual cycle (e.g., duration and regularity of the menstrual cycle) and to the PD characteristics (e.g., symptoms and pain intensity). The questionnaire is in Supplementary file 1.

The 11-point numerical rating scale for pain was used to assess the intensity of PD. This instrument ranges from zero to ten, with zero meaning absence of pain and 10 indicating the greatest pain the participant has ever felt. The scale is easily administered and simple to score, and its test-retest reliability is moderate to high, ranging from 0.67 to 0.96 [34]. This instrument is already been validated to assess PD in the Brazilian population [35]. The PD intensity was classified according to the scores obtained by the numerical rating scale for pain, considering the pain intensity as mild (1 to 3), moderate (4 to 7), or severe (score greater than or equal to 8) [36, 37]. The questionnaire used during data collection had 3 different questions about PD intensity. In each of them, participants had to fill out the scale considering three different time points: (I) PD intensity during the previous menstruation, (II) pain average for the three previous menstruations, and (III) the average of PD during the five previous years. The scale was applied only once, and participants were encouraged to recall the pain intensity during the three different time points.

### Statistical analysis

Data were coded and analyzed using the Statistical Package for the Social Sciences (SPSS) software version 23. Continuous and categorical variables were analyzed by frequency and descriptive analyses. The concordance between the pain intensity of the three time points (1 and 3 months and five years prior to their response) was assessed by the Kappa linear concordance test and classified as none to mild (0-0.20), regular (0.21–0.40), moderate (0.41–0.60), substantial (0.61–0.80) and almost perfect (0.81-1.00) [38].

A binary logistic regression was performed considering the binary logistic regression method for variables that reached the *p* < 0.05 in bivariate analysis between groups. We presented the variables according to the 95% confidence interval (95%CI), considering the associated PD-related factors according to the moderate and severe pain intensity three months before participation. The Chi-square test was applied to identify differences between pain intensity, PD symptoms, and socio-demographic characteristics. A significance level of 5% was considered.

## Results

A total of 11,591 women completed the questionnaire. However, 10,070 participants were included in the final statistical analysis due to the exclusion of participants who were aged under 18 years (*n* = 400), reported any health condition associated with secondary dysmenorrhea (*n* = 337), did not menstruate in the previous year (*n* = 605) or completed the questionnaire twice (*n* = 179).

Table 1 refers to the participants’ sociodemographic, gynecological, and obstetric characteristics. The average age of the participants was 25.2 ± 6.4 years, ranging from 18 to 54 years. About 19% of the participants were married, and 88% had more than 11 years of schooling. Regarding the obstetric and gynecological history of the participants, 84.7% of the women were nulliparous, 17.2% reported not having a regular menstrual cycle, 34% used hormonal contraceptives, and 69.2% used medication to relieve PD.

**Table 1:** Participants’ sociodemographic, gynecological and obstetric characteristics

Variables	Category	Frequency (%)
Age (years) *n* = 10,058	18–23	5205 (51.7)
	24–39	4410 (43.8)
	40–54	443 (4.5)
Brazilian geographic region *n* = 10030	North	389 (3.9)
	North East	1481 (14.8)
	Midwest	593 (5.9)
	Southeast	5870 (58.5)
	South	1697 (16.9)
Marital Status *n* = 10028	Married or cohabiting	1900 (19.0)
	Single or divorced or widow	8128 (81.0)
Scholarly *n* = 10061	Up to 8 years	32 (0.3)
	Between 9 and 11	1167 (11.6)
	More than 11	8862 (88.1)
Previous pregnancies *n* = 10054	None	8530 (84.8)
	1	866 (8.7)
	2	444 (4.4)
	3 or more	214 (2.1)
Type of birth *n* = 1313	Vaginal	420 (32.0)
	Caesarean	791 (60.2)
	Vaginal and caesarean	102 (7.8)
Age of menarche *n* = 9943	≤ 10 years	1211 (12.2)
	11 years	2686 (27.0)
	12 years	2899 (29.2)
	13 years	1875 (18.8)
	14 years	910 (9.1)
	≥ 15 years old	362 (3.7)
Duration of the menstrual cycle *n* = 9946	Irregular	1732 (17.2)
	Less than 28 days	1634 (16.5)
	28–29 days	4508 (45.4)
	30–31 days	1487 (15.0)
	More than 32 days	585 (5.9)
Use of hormonal contraceptives *n* = 8999	Yes	3421 (38.0)
	No	5578 (62.0)
Medicine for dysmenorrhea *n* = 9932	Yes	6967 (70.1)
	No	2965 (29.9)

The PD intensity was moderate, and the prevalence for the 1st and 3rd months and five years of last menstruation was 40.4%, 41.9%, and 49.7%, respectively. Severe pain was reported by 21.2% in the last menstrual cycle, 24.8% in the previous three menstrual cycles, and 28.4% in the last five years. The PD intensity for the three time points is presented in Table 2. Substantial agreement was found between the self-reported pain intensity for the 1-month and 3-month timepoints (κ = 0.68); and 1-month and 5-year timepoints (κ = 0.62). The agreement between pain intensity for the 3-month and 5-year timepoints was moderate (κ = 0.51). These findings indicated that PD could be deemed persistent in the present study.

**Table 2:** Pain intensity related to the PD in the last cycle, in the last three cycles and in the last five years (*n* = 10.070)

Pain intensity	In the previous menstrual cycle	In the previous 3 menstrual cycles	In the previous 5 years
Painless	926 (9.2)	614 (6.1)	239 (2.4)
Mild	2931 (29.1)	2488 (24.7)	1942 (19.3)
Moderate	4064 (40.4)	4223 (41.9)	5011 (49.7)
Severe	2133 (21.2)	2503 (24.8)	2861 (28.4)

Table 3 shows the intensity of symptoms related to the menstrual cycle. More than 30% of study participants reported that symptoms of irritability (44.9%), abdominal distension sensation (31.4%), anxiety (31.4%), and feeling more emotional (32.0%) were intense during menstruation.

**Table 3:** Prevalence and intensity of symptoms related to the menstrual period, presented as n (%)

Symptoms	Absent	Mild	Moderate	Severe
Irritability (*n* = 9889)	394 (4.0)	1515 (15.3)	3538 (35.8)	4442 (44.9)
Abdominal distension sensation (*n* = 9780)	796 (8.1)	2196 (22.5)	3721 (38.0)	3067 (31.4)
Sickness (*n* = 9649)	1086 (11.2)	2883 (29.9)	3491 (36.2)	2189 (22.7)
Breast pain (*n* = 9798)	1361 (13.9)	2623 (26.7)	3426 (35.0)	2388 (24.4)
Acne or dermatological problems (*n* = 9760)	1635 (16.8)	3126 (32.0)	3029 (31.0)	1970 (20.2)
Anxiety (*n* = 9701)	1683 (17.4)	2044 (21.1)	2923 (30.1)	3051 (31.4)
More emotional (*n* = 9643)	1751 (18.1)	2136 (22.2)	2669 (27.7)	3087 (32.0)
Appetite change (*n* = 9532)	2405 (25.2)	2090 (22.0)	2865 (30.0)	2172 (22.8)
Headache *n* = 9695	2411 (24.9)	2642 (27.3)	2799 (28.8)	1843 (19.0)
Posterior lumbar/pelvic pain (*n* = 9632)	2625 (27.3)	2094 (21.7)	2653 (27.5)	2260 (23.5)
Difficulty to concentrate (*n* = 9464)	3013 (31.8)	2592 (27.4)	2378 (25.1)	1481 (15.7)
Diarrhea (*n* = 9460)	3651 (38.6)	3033 (32.1)	2166 (22.9)	610 (6.4)
Lower limb pain (*n* = 9496)	4444 (46.8)	1862 (19.6)	1747 (18.4)	1443 (15.2)
Decreased sleep quality (*n* = 9429)	4576 (48.5)	2353 (25.0)	1652 (17.5)	848 (9.0)
Nausea (*n* = 9344)	5270 (56.4)	2389 (25.6)	1299 (13.9)	386 (4.1)
Joint pain (*n* = 9441)	5450 (57.7)	1816 (19.3)	1331 (14.1)	844 (8.9)

The results from the association and regression analysis are shown in Table 4. The Chi-squared test was significant for all the variables included (*p* < 0.05 for all analyses). Women with moderate menstrual pain intensity are young (18–23 years), nulliparous, presented PD since adolescence, complained about headache with moderate to severe intensity, mild diarrhea, moderate to severe sickness, irritability, appetite change, sensation of abdominal bloating, breast pain, feeling more emotional, difficulty to concentrate, increased anxiety and low back pain.

**Table 4:** Factors associated with pain intensity related to PD in the last 3 menstrual cycles

Variables	Participants’ answers	Absence or Mild PD *n* (%)	Moderate PD *n* (%)	Severe PD *n* (%)	Total	*p*-value #	OR (95%CI) Moderate PD	OR (95%CI) Severe PD
**Age**	18–23 years old	1488 (15.2)	2247 (22.9)	1310 (13.3)	5045 (51.4)	< 0.01	**1.4 (1.1–1.8)**	1.2 (0.9–1.6)
	24–39 years old	1455 (14.8)	1802 (18.4)	1075 (10.9)	4332 (44.1)		1.2 (0.9–1.5)	1.0 (0.8–1.3)
	40–54 years old	158 (1.6)	167 (1.7)	114 (1.2)	439 (4.5)		1.0	1.0
**Gestation**	No	2536 (25.8)	3627 (37.0)	2150 (21.9)	8313 (84.7)	< 0.01	**1.4 (1.2–1.5)**	**1.4 (1.2–1.6)**
	Yes	564 (5.7)	591 (6.0)	345 (3.5)	1500 (15.3)		1.0	1.0
**Dysmenorrhea since adolescence**	No	2114 (21.5)	1400 (14.2)	576 (5.9)	4090 (41.6)	< 0.01	1.0	1.0
	Yes	988 (10.0)	2823 (28.7)	1927 (19.6)	5738 (58.4)		**4.3 (3.9–4.8)**	**7.2 (6.3–8.1)**
**Headache**	Absent	990 (10.5)	908 (9.6)	442 (4.7)	2340 (24.7)	< 0.01	1.0	1.0
	Mild	864 (9.1)	1122 (11.9)	600 (6.3)	2586 (27.3)		**1.4 (1.2–1.6)**	**1.6 (1.4–1.8)**
	Moderate/severe	1123 (11.9)	2036 (21.5)	1374 (14.5)	4533 (47.9)		**2.0 (1.8–2.2)**	**2.7 (2.4–3.1)**
**Diarrhea**	Absent	1338 (14.5)	1472 (15.9)	756 (8.2)	3566 (38.6)	< 0.01	1.0	1.0
	Mild	936 (10.1)	1311 (14.2)	716 (7.8)	2963 (32.1)		**1.3 (1.1–1.4)**	**1.3 (1.2–1.5)**
	Moderate/severe	636 (6.9)	1196 (13.0)	867 (9.4)	2699 (29.2)		**1.7 (1.5–1.9)**	**2.4 (2.1–2.8)**
**Nausea**	Absent	2030 (22.3)	2122 (23.3)	964 (10.6)	5116 (56.1)	< 0.01	1.0	1.0
	Mild	574 (6.3)	1121 (12.3)	647 (7.1)	2342 (25.7)		**1.9 (1.7–2.1)**	**2.4 (2.1–2.7)**
	Moderate/severe	251 (2.7)	688 (7.5)	715 (7.8)	1654 (18.1)		**2.6 (2.2–3.1)**	**6.0 (5.1–7.1)**
**Sickness**	Absent	562 (6.0)	382 (4.1)	120 (1.3)	1064 (11.3)	< 0.01	1.0	1.0
	Mild	1092 (11.6)	1208 (12.8)	514 (5.5)	2814 (29.9)		**1.6 (1.4–1.9)**	**2.2 (1.8–2.8)**
	Moderate/severe	1276 (13.5)	2480 (26.3)	1781 (18.9)	5537 (58.8)		**2.9 (2.5–3.3)**	**6.5 (5.3–8.1)**
**Irritability**	Absent	194 (2.0)	123 (1.3)	72 (0.7)	389 (4.0)	< 0.01	1.0	1.0
	Mild	649 (6.7)	564 (5.8)	272 (2.8)	1485 (15.4)		**1.4 (1.1–1.8)**	1.1 (0.8–1.5)
	Moderate/severe	2184 (22.6)	3475 (36.0)	2117 (21.9)	7776 (80.6)		**2.5 (2.0–3.2)**	**2.6 (2.0–3.4)**
**Appetite change**	Absent	967 (10.4)	950 (10.2)	423 (4.5)	2340 (25.2)	< 0.01	1.0	1.0
	Mild	690 (7.4)	880 (9.5)	461 (5.0)	2031 (21.8)		**1.3 (1.1–1.5)**	**1.5 (1.3–1.8)**
	Moderate/severe	1258 (13.5)	2181 (23.4)	1490 (16.0)	4929 (53.0)		**1.8 (1.6–2.0)**	**2.7 (2.4–3.1)**
**Sensation of abdominal bloating**	Absent	346 (3.6)	308 (3.2)	120 (1.3)	774 (8.1)	< 0.01	1.0	1.0
	Mild	891 (9.3)	824 (8.6)	416 (4.4)	2131 (22.3)		1.0 (0.9–1.2)	**1.3 (1.1–1.7)**
	Moderate/severe	1752 (18.4)	2992 (31.4)	1892 (19.8)	6636 (69.5)		**1.9 (1.6–2.3)**	**3.1 (2.5–3.9)**
**Breast pain**	Absent	594 (6.2)	511 (5.3)	226 (2.4)	1331 (13.9)	< 0.01	1.0	1.0
	Mild	972 (10.2)	1062 (11.1)	530 (5.5)	2564 (26.8)		**1.3 (1.1–1.5)**	**1.4 (1.2–1.7)**
	Moderate/severe	1427 (14.9)	2557 (26.7)	1683 (17.6)	5667 (59.3)		**2.1 (1.8–2.4)**	**3.1 (2.6–3.7)**
**Decrease in sleep quality**	Absent	1795 (19.5)	1856 (20.2)	803 (8.7)	4454 (48.4)	< 0.01	1.0	1.0
	Mild	615 (6.7)	1022 (11.1)	658 (7.1)	2295 (24.9)		**1.6 (1.4–1.8)**	**2.4 (2.1–2.7)**
	Moderate/severe	466 (5.1)	1085 (11.8)	897 (9.7)	2448 (26.6)		**2.2 (2.0–2.5)**	**4.3 (3.7–4.9)**
**More emotional**	Absent	250 (2.6)	154 (1.6)	101 (1.0)	505 (5.3)	< 0.01	1.0	1.0
	Mild	677 (7.1)	649 (6.8)	311 (3.2)	1637 (17.1)		**1.6 (1.2–1.9)**	1.1 (0.9–1.5)
	Moderate/severe	2092 (21.8)	3327 (34.7)	2029 (21.2)	7448 (77.7)		**2.6 (2.1–3.2)**	**2.4 (1.9–3.0)**
**Difficulty to concentrating**	Absent	1268 (13.7)	1150 (12.5)	509 (5.5)	2927 (31.7)	< 0.01	1.0	1.0
	Mild	781 (8.5)	1171 (12.7)	581 (6.3)	2533 (27.4)		**1.6 (1.5–1.9)**	**1.8 (1.6–2.1)**
	Moderate/severe	835 (9.0)	1666 (18.0)	1268 (13.7)	3769 (40.8)		**2.2 (2.0–2.5)**	**3.8 (3.3–4.3)**
**Increased anxiety**	Absent	747 (7.9)	613 (6.5)	271 (2.9)	1631 (17.2)	< 0.01	1.0	1.0
	Mild	753 (8.0)	834 (8.8)	403 (4.3)	1990 (21.0)		**1.3 (1.2–1.6)**	**1.5 (1.2–1.8)**
	Moderate/severe	1467 (15.5)	2629 (27.8)	1748 (18.5)	5844 (61.7)		**2.2 (1.9–2.5)**	**3.3 (2.8–3.8)**
**Pain in lower limbs**	Absent	1702 (18.4)	1786 (19.3)	821 (8.9)	4309 (46.5)	< 0.01	1.0	1.0
	Mild	568 (6.1)	802 (8.7)	450 (4.9)	1820 (19.6)		**1.3 (1.2–1.5)**	**1.6 (1.4–1.9)**
	Moderate/severe	628 (6.8)	1398 (15.1)	1107 (11.9)	3133 (33.8)		**2.1 (1.9–2.4)**	**3.6 (3.2–4.2)**
**Low back pain**	Absent	1137 (12.1)	1009 (10.7)	396 (4.2)	2542 (27.1)	< 0.01	1.0	1.0
	Mild	760 (8.1)	858 (9.1)	428 (4.6)	2046 (21.8)		**1.3 (1.1–1.4)**	**1.6 (1.4–1.9)**
	Moderate/severe	1033 (11.0)	2181 (23.2)	1592 (16.9)	4806 (51.2)		**2.4 (2.1–2.7)**	**4.4 (3.8–5.1)**
**Joint pain**	Absent	2058 (22.3)	2217 (24.1)	1005 (10.9)	5280 (57.3)	< 0.01	1.0	1.0
	Mild	460 (5.0)	821 (8.9)	498 (5.4)	1779 (19.3)		**1.7 (1.5–1.9)**	**2.2 (1.9–2.6)**
	Moderate/severe	358 (3.9)	941 (10.2)	848 (9.2)	2147 (23.3)		**2.4 (2.1–2.8)**	**4.8 (4.2–5.6)**

# *p*-value referring to Chi-Square test; this test was applied in order to analyze if there was any difference between the severity of the symtopms among the three groups

Symptoms that seem to be associated with the increased risk for moderate PD intensity are age between 18 and 23 years (OR 1.4, 95%CI 1.1–1.8), mild irritability (OR 1.4, 95% CI 1.1–1.8), moderate/severe sensation of abdominal bloating (OR 1.9; 95% CI 1.6–2.3) and mild sensation of being more emotional (OR 1.6; 95% CI 1.2–1.9). Severe PD intensity is associated with mild sensation of abdominal bloating (OR 1.3, 95% CI 1.1–1.7). The risk for both moderate and severe pain intensity related to PD seems to increase in nulliparous (moderate PD: OR 1.4; 95%CI 1.2–1.5; severe PD: OR 1.4; 95%CI 1.2 1.6), women with PD since adolescence (moderate PD: OR 4.3, 95% CI 3.9–4.8; severe PD: OR 7.2; 95% CI 6.3–8.1), with mild headaches (moderate PD: OR 1.4, 95% CI 1.2–1.6; severe PD: OR 1.6, 95% CI 1.4–1.8), mild diarrhoea, moderate/severe diarrhoea, mild nausea, mild sickness, moderate/severe sickness, mild appetite change, moderate/severe appetite change, mild breast pain, moderate/severe breast pain, moderate/severe breast pain, mild decrease in sleep quality, moderate/severe decrease in sleep quality, mild difficulty to concentrate, mild increased anxiety, moderate/severe increased anxiety, mild pain in lower limbs, moderate/severe pain in lower limbs, mild low back pain, moderate/severe low back pain; mild joint pain; moderate/severe joint pain with moderate/intense irritability, sensation of abdominal distension and feeling more emotional. Data about the *p*-value and 95%CI are shown in Table 4.

## Discussion

The main findings of the present study showed that the prevalence of PD is high among Brazilian women, with moderate PD-related pain intensity in the three-time points; this also points to the fact that PD could be deemed persistent in the present study. Moreover, our results indicated that the intensity of the symptoms associated with the menstrual cycle could increase the risk for moderate to severe menstrual pain.

We found a high overall prevalence of PD among Brazilian women, varying from 90.7% during the last menstrual cycle, 91.4% during the previous three cycles, and 97.4% during the last five years, considering mild, moderate, and severe symptoms. The results of the present study are similar to the previous literature that already reported a high PD prevalence among women worldwide [17–30], especially to the high percentages reported by studies conducted in Ireland (91.5%) [18], Malaysia (89.1%) [22], South Arabia (80.1%) [22] and France (79%) [19]. However, these percentages are higher when compared with countries from North America (60% in Canada [26], 64% in Mexico [27]) and Asia (41.7% and 51.1% in China [28, 29]). These could be related to the cultural characteristics and the methods used for data collection. Similar results were found by Chen et al. [28]; authors associated this variation with the dissimilarity in the definitions of PD, data collection methods, and study population.

Participants included in the present study were encouraged to assess their menstrual pain intensity considering three different time points (previous menstrual cycle, the previous three months, and the last five years), and moderate and substantial agreement were found between the perception of PD intensity according to the three different time points analyzed. These findings indicated that PD is persistent for most participants in this study. It is known that individuals with chronic pain showed a more reliable memory associated with the memory of pain, even after one year of the episodes. Therefore, health professionals should assess and consider the individuals’ painful memories, as well as the cognitive, affective, and motivational influences of the pain, as it is expected that preventive activities or treatment sessions could reduce the probability of developing new painful memories [39].

The most common symptoms associated with PD in Brazilian women included emotional and mental symptoms (irritability, anxiety, feeling emotional, appetite change, difficulty concentrating, decreased sleep quality) as well as physical complaints (abdominal bloating sensation, sickness, breast pain, acne or dermatological problems, headache, posterior lumbar or pelvic pain, diarrhea, lower limb pain, nausea, and joint pain). In addition, our study highlighted that the severity of the symptoms could increase the risk of moderate/severe pain intensity related to PD.

Previous studies have described the association between pain, irritability, fatigue, mood and appetite changes, and discomfort [25, 28, 40]. Although we believe that cultural and social influences may interfere with the results reported by different studies conducted in different countries, the main symptoms associated with PD in the present study are similar to previous studies conducted in other countries. These results should concern health professionals and public institutions worldwide, as many women residing in different places could be affected by symptoms associated with PD.

Moreover, findings from this study also highlighted that personal characteristics, as age between 18 and 23 years, the presence of PD since adolescence, and nulliparity are associated with PD-related pain intensity. These results follow the previous literature, especially regarding age, as authors reported a higher prevalence of PD in younger women [8, 9, 21, 40].

Therefore, there is a need to assess the symptoms reported by women with PD and plan strategies to control symptoms associated with PD and strategies to prevent this condition, considering the influence of personal factors and the prevalence of PD. Educational strategies would possibly make women aware of PD and its associated symptoms and the risk of presenting pain with moderate to severe intensity during the menstrual cycle. Simple tools, such as booklets, are already validated and available in Brazil and could be helpful tools during educational activities conducted by health professionals [41]. Conservative techniques could relieve the pain intensity of PD and symptoms highly associated with the menstrual cycle. Some strategies, such as physical exercise [42], the use of topical heat [43], acupuncture [44], and transcutaneous electrical stimulation [45] are already associated with the management of PD-related pain and symptoms. Those strategies should be used in the management of this condition, especially considering that women with PD have lower quality of life [46], and may have their daily activities affected by the PD, which is also associated with a decline in women’s productivity [10, 47] and a high absenteeism rate from school and work [13, 14, 47].

This study has some limitations. The present study was conducted during the COVID-19 pandemic, while presential activities were canceled and remote activities were conducted for most research laboratories [48]. Another limitation is the possibility of women with PD being more engaged in answering the questionnaire, as women with pain would be more interested in the current research topic. Moreover, participants included in this survey may present undiagnosed secondary dysmenorrhea. However, to identify participants with symptoms of secondary dysmenorrhea, all participants were asked about diseases associated with secondary symptoms (i.e., endometriosis and myoma) and excluded in case of an affirmative answer.

On the other hand, the present study has several strengths. To the authors’ knowledge, this is the first study to report a drawn profile of the prevalence and associated factors of PD in Brazilian women nationwide. Although previous studies already reported the prevalence of PD in the Northeast part of Brazil [31, 32], authors included only university women (aged about 25 years old) in their sample [31]. It seems that the perception of women about PD can vary depending on the women’s context, including different age groups [49]. Therefore, women with varying age ranges should be included in populational studies to report more pragmatic results. In the present study, the age range of the participants was from 18 to 54 years old.

As this study was conducted remotely, reaching the entire national territory was possible, ensuring a greater number of participants and a better representation of the Brazilian population. Moreover, the study sample size was important (10,070 valid responses included in the data analysis). The present results could help design health strategies to reduce PD symptoms’ impact on women’s lives and improve their quality of life, along with public policies regarding health promotion and prevention of PD-related pain and symptoms.

## Conclusion

There is a high prevalence of PD among Brazilian women considering three different timepoints. The most common symptoms associated with PD are irritability, abdominal distension sensation, sickness, breast pain, acne or dermatological problems, anxiety, feeling emotional, appetite change, headache, posterior lumbar or pelvic pain, difficulty concentrating, diarrhea, lower limb pain, decreased of sleep quality, nausea, and joint pain.

The intensity of symptoms related to the menstrual cycle is associated with increased odds of moderate to severe pain intensity related to PD. The increased risk for moderate PD intensity is related to age between 18 and 23 years and mild irritability. Mild sensation of abdominal distension increases the risk for severe pain intensity associated with PD. The risk for both moderate and severe PD- related pain intensity seems to increase in nulliparous women, women with PD since adolescence, with mild or moderate and intense headaches, diarrhea, nausea, sickness, appetite change, breast pain, decrease in sleep quality, difficulty to concentrate, increased anxiety, lower limbs, low back, and joint pain; with moderate/intense irritability, sensation of abdominal distension and feeling more emotional.

### Electronic supplementary material

Below is the link to the electronic supplementary material.

Supplementary Material 1

## Data Availability

The datasets generated and analysed during the current study are not publicly available due to personal content that can lead to participant identification but are available from the corresponding author on reasonable request.
