# Supplementary material for: Prevalence, pain intensity and symptoms associated with primary dysmenorrhea: a cross-sectional study
Source: BMC Womens Health. 2024 Feb 4;24:92. doi: 10.1186/s12905-023-02878-z (PMC10840141; doi:10.1186/s12905-023-02878-z)
Supplement: Supplementary file 1 — Supplementary Material 1 [file 12905_2023_2878_MOESM1_ESM.pdf]

1. Did you menstruate in the past 3 months?

( ) Yes

( ) No

2. In which Brazilian state do you currently live in?

(List of Brazilian states)

3. In which city do you live?

(short answer)

4. Marital status

( ) Single

( ) Married

( ) Widow

( ) Divorced

5. Scholarity

( ) Up to 8 complete years

( ) 9 to 11 complete years

( ) more than 11 complete years

6. How old were you when you first menstruated?

( ) ≤ 10 years old

( ) 11 years old

( ) 12 years old

( ) 13 years old

( ) 14 years old

( ) 15 years old

( ) ≥ 16 years old

7. What is the MEAN duration of your menstrual cycle?

( ) less than 27 days

( ) 28-29 days

( ) 30-31 days

( ) over 31 days

( ) I have irregular menstrual cycles

( ) I don’t know / I don’t want to answer

8. How many days does your menstruation last?

( ) less than 3 days

( ) 3 days

( ) 4 days

( ) 5 days

( ) 6 or more days

( ) I don’t know / I don’t want to answer

9. Have you started your sexual life?

( ) Yes

( ) No

( ) I don’t know / I don’t want to answer

10. Do you use any form of contraceptive at this present moment?

( ) Copper intrauterine device

( ) Hormonal intrauterine device

( ) Hormonal injection

( ) Oral contraceptive

( ) Male / female condom

( ) Implants

( ) I don’t use any contraceptive methods

( ) I don’t know / I don’t want to answer

10.1. How long have you been using this method?

( ) less than a month
( ) 1-2 months
( ) 3-6 months
( ) 7-12 months
( ) over a year
( ) I don’t know / I don’t want to answer

10.2. This chosen methods was medically prescribed?

( ) Yes
( ) No
( ) I don’t know / I don’t want to answer

10.3. If medically prescribed, did you receive enough information on the method and its side effects?

( ) Yes
( ) No
( ) I don’t know / I don’t want to answer

11. How many pregnancies did you have?

( ) None

( ) 1

( ) 2

( ) 3

( ) 4

( ) 5 or more

( ) I don't know / I don't want to answer

12. Have you ever had an abortion?

( ) Yes

( ) No

( ) I don't know / I don't want to answer

13. Have you ever been pregnant with twins?

( ) Yes

( ) No

( ) I don't know / I don't want to answer

14. How many children do you have?

( ) None

( ) 1

( ) 2

( ) 3

( ) 4 or more

( ) I don't know / I don't want to answer

15. What kind of birth did you experience?

( ) None

( ) Vaginal

( ) Cesarean

( ) Vaginal and cesarean

( ) I don't know / I don't want to answer

16. Are you breastfeeding right now?

( ) Yes

( ) No

17. On a scale of 0 to 10, with 0 being no pain and 10 being the worst pain you can imagine, how would you rate the pain you feel during your menstrual cramps? Answer the next three questions based on this scale.

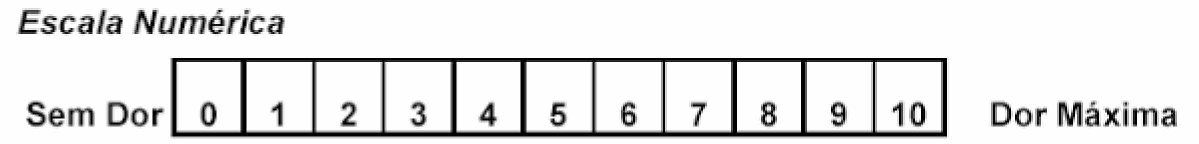

17.1. What is the mean pain intensity of your menstrual cramps in the past 5 years?

(list of 0-10)

17.2. Regarding the past month, what was your menstrual pain?

(list of 0-10)

17.3. Regarding the past three months, what was your menstrual pain?

(list of 0-10)

18. Which of the options below is true for you? (You may mark more than one option)

( ) I have had menstrual cramps since my adolescence.

( ) My menstrual cramps are more or less similar all months.

( ) My menstrual pain intensity has decreased over the past years.

( ) My menstrual pain intensity has increased over the past years.

( ) From my child’s birth, my menstrual pain intensity increased.

( ) From my child’s birth, my menstrual pain intensity decreased.

( ) I have never had menstrual cramps.

( ) I rarely have menstrual cramps.

( ) When I am more stressed, I have more pain related to my menstrual cramps.

19. Which symptoms do you associate with your pre-menstrual (days before your menstruation) and menstrual periods?

( ) Cramps	(I don’t have)	(mild)	(moderate)	(severe)
( ) Headache / migraine	(I don’t have)	(mild)	(moderate)	(severe)
( ) Diarrhea	(I don’t have)	(mild)	(moderate)	(severe)
( ) Nausea	(I don’t have)	(mild)	(moderate)	(severe)
( ) Indisposition	(I don’t have)	(mild)	(moderate)	(severe)
( ) Irritability	(I don’t have)	(mild)	(moderate)	(severe)
( ) Appetite change	(I don’t have)	(mild)	(moderate)	(severe)

( ) Abdominal distension	(I don’t have)	(mild)	(moderate)	(severe)
( ) Breast swelling	(I don’t have)	(mild)	(moderate)	(severe)
( ) Lower limbs swelling	(I don’t have)	(mild)	(moderate)	(severe)
( ) Decrease on sleep quality	(I don’t have)	(mild)	(moderate)	(severe)
( ) Skin problems such as acne	(I don’t have)	(mild)	(moderate)	(severe)
( ) Dizziness	(I don’t have)	(mild)	(moderate)	(severe)
( ) Fainting	(I don’t have)	(mild)	(moderate)	(severe)
( ) Ringing sensation in the ear	(I don’t have)	(mild)	(moderate)	(severe)
( ) More emotive than usual	(I don’t have)	(mild)	(moderate)	(severe)
( ) Concentration difficulty	(I don’t have)	(mild)	(moderate)	(severe)
( ) Anxiety	(I don’t have)	(mild)	(moderate)	(severe)
( ) Low self-esteem	(I don’t have)	(mild)	(moderate)	(severe)
( ) Lower limbs pain	(I don’t have)	(mild)	(moderate)	(severe)
( ) Low back pain	(I don’t have)	(mild)	(moderate)	(severe)
( ) Joint pain	(I don’t have)	(mild)	(moderate)	(severe)

20. Do you have any of the medical diagnosis presented?

( ) Endometriosis

( ) Polycystic ovary

( ) Myoma

( ) Cervical cancer

( ) Vulvar edema

( ) Urogynecological infection or inflammation

( ) Genital malformation

( ) Uterus prolapse

( ) Vulvodinia

( ) Adenomyosis

( ) I do not have any of those diagnosis

( ) I don’t know / I don’t want to answer

21. Do you use any medication of continuous use?

( ) Yes

( ) No

( ) I don’t know / I don’t want to answer

21.1. In affirmative case, what kind of medication is it?

( ) Anxiolytic

( ) Antidepressive

( ) Contraceptive

( ) I don’t know / I don’t want to answer

22. Do you use pain killers for your menstrual pain?

( ) Yes

( ) No

( ) I don’t know / I don’t want to answer

22.1. In affirmative case, which medications do you use?

(short answer)

22.2. For how many days do you use those medications?

( ) 1

( ) 2

( ) 3

( ) 4

( ) 5

( ) All days during my menstruation

22.3. After using those medications, the pain:

( ) Does not change

( ) Has a mild improvement

( ) Has a large improvement

( ) Is completely resolved.
